# Risk Factors, Clinical Consequences, Prevention, and Treatment of Childhood Obesity

**DOI:** 10.3390/children9121975

**Published:** 2022-12-16

**Authors:** Mossad Abdelhak Shaban Mohamed, Merna Mahmoud AbouKhatwa, Abdul Aziz Saifullah, Muhammad Hareez Syahmi, Mohamed Mosaad, Mahmoud E. Elrggal, Inderpal Singh Dehele, Mohamed Hassan Elnaem

**Affiliations:** 1 Department of Pediatrics, Faculty of Medicine, International Islamic University Malaysia, Kuantan 25200, Malaysia; 2 Department of Clinical Pharmacy and Pharmacy Practice, Faculty of Pharmacy, Alexandria University, Alexandria 5372066, Egypt; 3 Faculty of Medicine, International Islamic University Malaysia, Kuantan 25200, Malaysia; 4 Faculty of Medicine, Widad University College, Kuantan 25200, Malaysia; 5 College of Pharmacy, Umm Al-Qura University, Makkah 21955, Saudi Arabia; 6 School of Pharmacy, University of Birmingham, Birmingham B15 2TT, UK; 7 School of Pharmaceutical Sciences, Universiti Sains Malaysia, George Town 11800, Malaysia

**Keywords:** risk factors, prevention, treatment, childhood, obesity

## Abstract

Obesity might adversely affect the health and well-being of children and their families. Childhood obesity has crucial implications for health, both during childhood and as they age. It is highly associated with many acute problems and is commonly present during childhood, making visits and hospital admissions polarized in this group of children. The problems that may affect these children can be medical, such as asthma, chronic inflammation, orthopedic abnormalities, liver disease, diabetes mellitus or dyslipidemia. Long-term consequences of cardiovascular risk factors, the persistence of obesity and premature mortality are common among adults who had obesity during their early lives. Additionally, they could also suffer from psychological issues, such as low self-esteem, which puts them at risk of a much more serious psychosocial problem that may lead to depression, as well as a disruption in educational achievements and social relationships. A healthy diet, physical activity, adequate sleep, and limited screen time are all preventive measures that should be implemented at the family and community levels, preferably through well-structured programs. Furthermore, pharmacological management of childhood obesity is limited and only used after non-pharmacological interventions have failed or in the late stages of obesity. However, recent guidelines advocate the early use of medical interventions. Approved pharmacotherapeutic options include orlistat, phentermine/topiramate combination and liraglutide. There are several other options approved primarily for other specific forms of obesity or for other indications, including setmelanotide, metformin, lisdexamfetamine, zonisamide and fluoxetine. Bariatric surgery is a safe and effective option in cases with extreme obesity and comorbidities considering the need for long-term monitoring and support for cases and their families post-surgery. This review aims to discuss and highlight the recent evidence regarding risk factors, clinical consequences, prevention, and treatment of childhood obesity.

## 1. Introduction

Obesity is an exorbitant build-up of adipose tissue that damages the well-being of an individual who suffers from that condition [1]. It is known to be caused by excess calorie intake in diets and inadequate energy consumption from physical activity, which can lead to a positive energy balance [2]. Various factors, such as genetic predispositions, behavior, and the environment, affect the risk of developing obesity [3]. Globally, millions of children under five years old are struck by having overweight and obesity [4]. The obesity prevalence among adolescents from 1975 to 2016 showed an increasing trend globally, with a 4.9% increment in girls and 6.9% in boys [5]. According to recent data, overweight and obesity affect approximately 330 million children and adolescents aged 5 to 19 years [6]. Childhood overweight and obesity have shown a rising trend within the past few decades worldwide, significantly in most high-income countries and, from the limited data accessible, rapidly in other income-class countries [7]. For example, in a systemic analysis from 1980 to 2013, the combined prevalence of overweight and obesity in children worldwide rose by 47.1% [8]. 

Childhood obesity is thought to increase the risk of developing various comorbidities, including hypertension, diabetes, precocious puberty, irregular menstruation, non-alcoholic fatty liver diseases, polycystic ovarian syndrome, sleep apnea, asthma, musculoskeletal and mental health diseases [9]. Therefore, following the preceding data, the rise in global obesity is a compelling health burden that should be addressed through a proper plan of action to withstand the impacts of childhood obesity upon our generations. This work aims to provide a comprehensive review of childhood obesity that discusses the contributing risk factors, the anticipated clinical consequences, prevention recommendations, and current treatment trends. 

## 2. Risk Factors

Although the cause of the incidence of obesity, in general, can be reduced to the problem of relatively disproportionate energy intake from diets and energy consumption by the body for daily usage, it is well-recognized that obesity development is multifactorial and correlates with the child’s developmental process [10]. Those factors include nutrition, socioeconomic status (SES), individual lifestyle, antenatal history, weight at birth, and genetic factors. 

### 2.1. Nutrition and Diet

A commonly associated risk factor with cases of childhood obesity is nutrition or the type of diet the children have in their daily life [10]. It has been reported that those consuming more than two times carbonated soft drinks and at least one fast food daily were at a substantially higher risk of being overweight or developing obesity. This is due to the high energy content in food and drinks, which results in a positive energy balance [11]. A study on school adolescents in Malaysia found that consuming dairy products and milk among adolescents was positively associated with obesity [12]. The quality of the diet is also associated with the risk of obesity and the correlation between dietary habits and the prevalence of overweight and obesity has been well-established in the literature [13]. Healthy Eating (HEI) study observed that obesity in adolescents correlated with diet quality [14]. Additionally, skipping breakfast is a critical risk factor for obesity development, as several studies reported a significant association between skipping breakfast and the risk of adiposity [15].

It is essential to highlight that the challenge of the overabundance of calories has several contributing factors. As energy-dense and ultra-processed foods become more readily available, they are designed to enhance the reward response by stimulating dopamine receptors in the brain [16]. Portion size is also critical considering the increase in sizes by food industries with the increasing trend of eating outside the home [17]. A study on dietary intake found that consuming vegetables at least twice per day can act as a protective measure against overweight and obesity [11]. Therefore, adherence to recommended food diets is critical for lowering the rate of overweight and obesity in children and adolescents.

### 2.2. Socioeconomic Factors

Childhood obesity is also influenced by socioeconomic factors, including low socioeconomic status, low parental education, non-parental caregivers, a lower fruit-eating frequency, short sleeping hours, and parental obesity [18]. Moreover, adverse childhood experiences are social determinants of health that increase the risk of the incidence of overweight and obesity in adolescence [19]. The lack of nutrition assistance programs and designated areas for active transportation and exercise creates variations and puts some members of the community with restricted access to such services, at a higher risk of obesity [20].

### 2.3. Comorbidities and Healthcare System-Related Factors

Concerning associated medical conditions, unhealthy weight was reported among children with comorbidities such as autism spectrum disorders and those with sleep and affective problems [21]. The lack of strategies to prevent weight stigma, and the shortage of competent trained healthcare professionals to provide optimal timely management of eating disorders are also critical risk factors [22]. Finally, despite being a well-established contributor to many clinical consequences and being recognized by the public to be a disease, obesity management is still not a priority in different health insurance plans, making the affordability of treatment and accessibility to care challenging [23].

### 2.4. Maternal-Related Factors

Research has shown that higher pre-pregnancy BMI directly correlates with childhood obesity [24]. It has also been found that parental BMI can substantially affect their children’s body weight [12]. It is thought that fetal growth contributes to the development of lean body mass later in life, making intrauterine life an important period for developing childhood obesity [25]. Similarly, small-for-gestational-age children are prone to accumulating fat mass, particularly in the abdominal area [25]. The main contributing factor, in this case, is the obesogenic environment that the children were brought up in, which will soon become habitual and inherently turn into a lifestyle that the offspring will adopt at a later age [26]. In addition, abdominal obesity is linked to metabolic syndrome, which includes dyslipidemia, hypertension, and abnormal glucose and insulin regulation [27]. Yang et al. concluded that parents’ BMI above normal value was strongly correlated with an elevated risk of metabolic syndrome in their kids [28]. Therefore, this might suggest why obesity can sometimes have a familial correlation as one of the risk factors.

A study examining the impact of birth weight on overweight and obesity in early school age concluded that the risk of being overweight or obese at an early age was higher in those with higher weight at birth [29]. There is also consistent evidence demonstrating a linear association between birth weight and BMI in later life [10]. Birth weight often correlates positively with body size and adiposity in later life [30]. A Malaysian study suggested that promoting a healthy lifestyle during pregnancy prevents excessive fetal weight that may lead to higher birth weight and make the offspring prone to develop overweight and obesity [12].

### 2.5. Physical Activity

The frequency of physical activity among children is another risk closely related to dietary intake. The lack of physical activity coupled with poor dietary choices is closely attributed to the positive energy balance that is widely recognized as the main culprit for the incidence of obesity [31]. Lack of physical activity and an excessively sedentary lifestyle are highly associated with the risk of developing overweight or obesity in adolescents due to lower body energy consumption to burn calories from dietary intake [11]. The opposite direction of this association also coincided when the finding showed that higher physical activity could lower the risk of obesity by 10% for each hour per day of moderate–vigorous exercise. One study in Malaysia concluded a significant association between media viewing and screen time, leading to relatively lower physical activity as part of the multifactorial plague causing the rise of childhood obesity [32].

### 2.6. Sleep Duration and Quality

Sleep duration and quality are deemed significant risk factors for childhood obesity. A meta-analysis conducted by Han et al. reported that an increased risk of childhood obesity is accompanied by short sleeping durations, especially in children less than 6 and 6–10 years old [33]. Additionally, decreased sleep timing was associated with adverse dietary intake behavior such as a decrease in the intake of fruits [34].

## 3. Clinical Consequences

Many consequences of obesity that were previously thought to be adult-dominated diseases are now affecting children [35]. Childhood obesity can lead to several short- and long-term cardiovascular, respiratory, orthopaedic, endocrinology, mental health and gastrointestinal/liver diseases. In this section, the major medical conditions associated with obesity are discussed [36].

### 3.1. Cardiovascular Disease

Childhood obesity has detrimental effects on the cardiovascular system, similar to those seen in adults. The most common cardiovascular risk factors associated with childhood obesity are hyperinsulinemia or insulin resistance, dyslipidemia, hypertension, ventricular defect, and endothelial function abnormalities [35]. Moreover, children with obesity are approximately three times more susceptible to hypertension than children without obesity [37].

### 3.2. Respiratory Disease

Obstructive sleep apnea (OSA) can cause failure to thrive, behavioral issues, decreased intellectual function, and an increased risk of cardiovascular morbidity, insulin resistance and weight gain in children [38]. OSA in children was previously thought to be only caused by adenotonsillar hypertrophy, but it has changed with the rise of childhood obesity [38]. Next, obesity certainly increases the risk of asthma, but the mechanism underlying this is not fully understood [39]. However, it has been known that obesity is more common in children with asthma, and studies of adults and children have found a link between asthma and a high body mass index (BMI) [40].

### 3.3. Endocrine Disease

The rising prevalence of childhood obesity has coincided with an increased incidence of T2DM in youths [36]. Furthermore, the earlier the onset of the disease, the greater the accelerated deterioration of the beta cell, making youths vulnerable to developing adult-like metabolic comorbidities at an early age [10]. The association between diabetes, impaired glucose tolerance and obesity is thought to be regulated through oxidized low-density lipoprotein antibodies [41].

Central precocious puberty (CPP) is a condition in which puberty begins before the age of 8 in girls and 9 in boys and is characterized by the early onset of secondary sexual characteristics due to the early onset of pulsatile secretion of gonadotrophin-releasing hormone [42]. Obesity is recognized as a significant underlying cause of CPP; where a study in China highlighted that being overweight and obese is related to increased odds of developing CPP, especially among girls [43]. Moreover, CPP was more likely to develop and occur when overweight or obesity persisted for more than one year in girls and two years in boys.

Moreover, an initial evaluation of whether girls with precocious adrenarche (PA) born at appropriate gestational age (AGA) were at greater risk of metabolic complications showed favorable lipid profiles upon metabolic screening and suggested that the atherogenic index increases with an increase in BMI and waist circumference in girls with PA [44].

Regarding the link between obesity and polycystic ovary syndrome (PCOS), there is some evidence that obesity might impact the risk of PCOS through insulin resistance and compensatory hyperinsulinemia, which increases ovarian/adrenal androgen production, as well as sex hormone-binding globulin (SHBG) suppression, which increases androgen bioavailability [45]. This demonstrates the link between peripubertal obesity and the increased prevalence and severity of adolescent PCOS.

### 3.4. Mental Health and Psychosocial

People with obesity are frequently subjected to public disapproval and stigma because of their weight, with women facing more discrimination [46]. In children with obesity, psychosocial issues can also arise as their weight is perceived as a significant handicap by others [35]. A study conducted in Sweden found that the odds of children with obesity getting anxiety and depression are higher than children without obesity [47]. Moreover, girls with obesity seemed to be at higher risk of getting anxiety and depression compared to boys with obesity [47]. Furthermore, girls and boys who are overweight or obese are at increased risk of bullying and academic failure, which creates a burden well into their adulthood [48].

### 3.5. Gastrointestinal and Hepatic Diseases

Childhood obesity significantly increases the risk of NAFLD among the pediatric population [49]. NAFLD manifests differently in children than in adults, with different biopsy findings and higher reported rates of fibrosis and cirrhosis [49]. Furthermore, according to a Chinese study, the incidence of NAFLD is high in obese children, and obesity is an important trigger of NAFLD, even though this disease can be found in a normal BMI population [50]. In addition, evidence showed an association between obesity and the risk of having gastroesophageal reflux disease, particularly among children 6–11 years and adolescents [51]. The prevalence of childhood obesity correlates with the increased hospitalization rates of pediatric cholelithiasis [52].

### 3.6. Infectious Diseases

Individuals who developed obesity are more prone to infections compared to those with normal weight. For example, data showed that individuals with obesity were more prone to exhibit Helicobacter pylori infections compared with those with normal BMI [53]. Obesity is associated with impaired immune responses and diminished functions of several immune cells, as there is a close interaction between immune tolerance and metabolic control [54]. The disruption of lymphoid tissue integrity can explain immune dysfunction in obese patients by fat accumulation and altered secretion of adipocytokines such as leptin or adiponectin [55]. 

### 3.7. Overall Increase in the Demand for Healthcare Services

As elaborated, obesity is linked to serious illnesses and health-related problems that may increase the demand for a range of health services and drug prescriptions. A recent Spanish longitudinal analysis highlighted that those children with obesity were more likely to demand health services for psychological and musculoskeletal conditions and consequently, they were more likely to receive an overall higher number of pharmacotherapy prescriptions [56]. A study conducted in England showed an increasing trend in hospital admission rates among children who had developed obesity, more significantly higher among girls than among boys [57]. Moreover, it was found that children with obesity are associated with prolonged hospitalization. In a study by Shanley et al. (2015), obese children admitted due to asthma have greater odds of prolonged length of hospital stay [58]. It is also complemented by another study that showed that children with obesity with severe asthma exacerbations have longer ICU and hospital lengths of stay [59].

### 3.8. Overall Increase in the Healthcare Costs

An analysis of the lifetime costs of childhood overweight and obesity uncovered a proportionality between body mass index and costs [60]. Compared to boys, girls have higher healthcare costs and income penalties, while boys have higher costs associated with lost workdays [60]. Medical expenditures on obesity have ranged from 5 to more than 12% of all medical expenditures across different states in the US [61]. A recent Italian study indicated that the total costs attributable to obesity amounted to EUR 13.34 billion in 2020 [62]. All these data underpin the direct and indirect burden imposed by obesity on the overall healthcare system.

## 4. Prevention

Despite the role of genetics, many other causative factors could be avoided to prevent pediatric obesity. In this section, the recent promising preventive approaches will be discussed. Implementing these interventions requires a societal approach that extends far beyond parental and educational directives. In fact, it should begin before becoming a parent or even sending children to school.

### 4.1. Obesogenic Environment-Oriented Approaches

Setting a healthy environment for infants requires multi-component intervention programs to reduce their risk of obesity. In addition, these approaches necessitate the participation of different parties, such as family, school, and community. Community-based strategies and socioeconomic factors play an influential role in combatting obesity in paediatrics. Governments (public sector) and food industries (private sector) share responsibility for reducing the obesogenic environment, as well as enforcing clear procedures and well-structured national programs to reduce the availability of highly processed foods in the food supply chain [63]. The range of preventive approaches may include banning the vending of calorie-rich beverages and food at schools, restricting the sale of these unhealthy obesogenic products, in the areas surrounding the schools, offering healthy but non-costly foods at schools and clubs, implementing taxation on junk food to limit their sale and spread in addition to encouraging students to be involved in a wide variety of physical activities at schools by providing playgrounds and gyms [15].

#### 4.1.1. Healthy Diet

Parents should support their children in making the right choices, appropriate timing and correct dietary proportions for better health and nutrition status. The United States Department of Agriculture (USDA) has provided an excellent practical example of these diet recommendations under the name “MyPlate,” which is an easy-to-follow food guide designed to assist parents in providing balanced and nutritious meals to their children [64]. It is a colorful divided plate with sections for vegetables (green), fruits (red), grains (orange), protein (purple), and dairy (blue). The My Plate website provides simple messages to guide parents in selecting items for a balanced meal, halving the plate with vegetables and fruits, providing at least half of the recommended grains as whole grains, serving fat-free or low-fat milk and water instead of sugary drinks, and emphasizing not serving oversized portions [64].

#### 4.1.2. Physical Activity

The child’s physical activity influence the risk of obesity. At least 60 min of moderate to vigorous exercise is recommended for children up to 15 years to combat obesity risk [65]. Promoting physical activity is not enough, but it should be combined with limiting screen time and a sedentary lifestyle [66].

#### 4.1.3. Screen Time

Screen time is associated with obesity development, and different media devices contribute differently to increasing obesity risk [67]. Therefore, the screen time limitation is considered a promising protective factor against childhood obesity. It is recommended that screen time should not exceed 2 h per day [66]. It might also be critical to acknowledge the impact of social media on the pattern of food consumption among a relatively younger population, particularly those who have tried losing weight or have developed obesity [68].

#### 4.1.4. Sleeping Duration and Quality

Maintaining adequate sleep duration and quality is essential for preventing the risk of developing overweight or obesity. Recommended sleeping duration ranges between 8 and 11 h in a quiet environment [66,69]. Adequate sleep duration is associated with increased consumption of healthier dietary choices of fruits and vegetables [70].

### 4.2. Maternal Health before and during Pregnancy

Alcohol consumption during pregnancy has detrimental effects on fetal and maternal gut microbiota composition. There was a significant correlation between maternal and neonatal well-being. Several studies documented maternal health influences on neonatal gut-microbiota composition, stating that infants born to obese women showed alterations in their gut-microbiota composition, increasing their risk of obesity [71]. In addition, placenta microbiota has also been shown to influence neonatal gut-microbiota composition [72]. Therefore, it is recommended to provide educational sessions to parents to encourage them to maintain a healthy diet before and during pregnancy and to quit smoking and alcohol consumption.

#### 4.2.1. Vaginal Delivery

According to a recent meta-analysis, the risk of offspring obesity is increased by 30% after cesarean section (C.S.) delivery compared to vaginal delivery. Although the exact underlying mechanism is unknown, it is assumed that gut microbiota is a contributing factor [73]. The role of vaginal delivery mode in decreasing the risk of obesity is insignificant if the mother is obese or overweight. A recent study reported that infants born vaginally or via cesarean delivery to mothers with obesity are at higher risk of obesity than those born to normal-weighted mothers [73]. So, maternal weight contributes markedly to the potential benefit of vaginal over C.S. delivery; thus, it is correlated with the mom’s BMI and weight gain during pregnancy.

#### 4.2.2. Breastfeeding

Compared to breastfeeding, formula feeding is associated with providing a greater level of fats and proteins, exceeding neonatal needs, eventually leading to an increased risk of obesity [74]. Breastfeeding is protective against pediatric adiposity, and its duration correlates with the risk of pediatric obesity [75]. For example, breastfeeding duration ≥7 months is linked to an approximately 21% decrease in the risk of obesity, while less duration for <3 months showed about a 10% decrease in the risk of childhood obesity [75]. Therefore, breastfeeding and its duration correlate well with decreasing the risk of childhood obesity, and thus, it represents the most appropriate feeding pattern for neonates. It is also essential to highlight the influence of the diet consumed by the infant after weaning, as it contributes substantially to healthy eating habits and the consumption of nutritious foods [76].

### 4.3. Strategies for Combating Weight Stigma and Enhancing Obesity Care

As part of the school-based curriculum, there should be strategies to help students prevent eating disorders and there should be school-based policies and social media campaigns designed to protect students from being bullied because of their weight [22]. Further, it is imperative to implement policies that recognize the clinical burden of obesity to make obesity management more accessible and affordable through the healthcare system and insurance companies.

#### Training of Healthcare Providers

**Figure 1:**
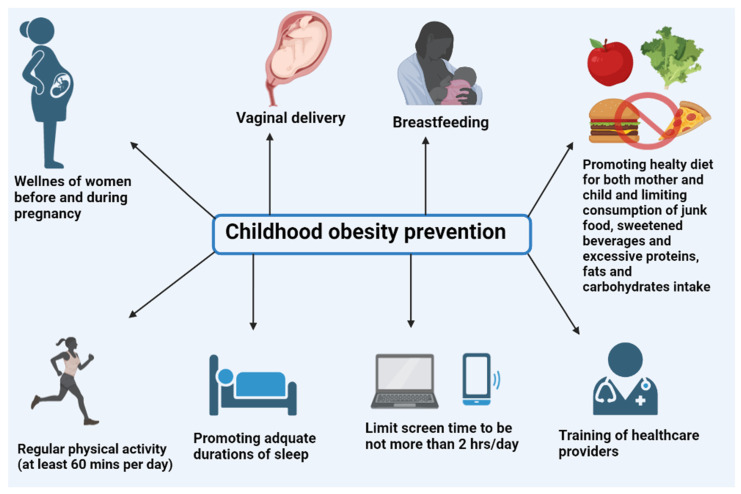
Childhood obesity prevention.

The lack of knowledge among primary care providers and specialists has been reported to be one of the most serious barriers to prevention practices implementation in addition to their deviation from guidelines and their reluctance to interfere if the child’s weight is identified to jump into overweight and/or obese range [77]. There is an urgent need for better integration of pediatric content training in the curricula to strengthen the academic knowledge and practical skills related to obesity prevention and treatment among newborns and infants [78]. In view of the growing burden of childhood obesity, there is still a need for capacity building and training for educators and healthcare providers to enable them to identify and manage disordered eating and abnormal weight gain at an early stage [22]. Figure 1 depicts the range of preventive strategies for childhood obesity.

## 5. Treatment

Anti-obesity medications are adjunctive treatment to dietary and physical activity changes in childhood obesity and are typically highly sought-after failure of non-pharmacological interventions or in the late stages of obesity [79]. However, recent guidelines advocate the early use of medical and surgical interventions [80].

### 5.1. FDA-Approved Medications

#### 5.1.1. Orlistat

Orlistat belongs to the lipase inhibitors pharmacological class approved in 1999 for managing obesity [81]. Its mechanism of action involves acting locally in the stomach and intestine, preventing triglyceride absorption by 30% and inhibiting the action of both gastric and pancreatic lipases [82]. Over a 54-week period, orlistat administration was associated with a significant BMI reduction of 0.55 compared to a BMI increase of 0.31 with placebo (*p* = 0.001) [83]. It has also been recommended as an adjunct therapy with optimized dietary intake and physical activity [84]. However, several adverse effects were documented, limiting the user preference for orlistat. Gastrointestinal side effects, including abdominal discomfort, pain, steatorrhea, and constipation, have been reported in addition to affecting the absorption of fat-soluble vitamins [84,85].

#### 5.1.2. Liraglutide

Liraglutide is an antidiabetic drug that belongs to the glucagon-like peptide 1 (GLP-1) receptor agonist class. It has emerged as a novel therapeutic drug that aids in weight loss by lowering energy intake and appetite and increasing fullness sensation. It also decreases glucagon secretion, delaying gastric emptying and elevation of post-prandial insulin levels. Its action is mediated through the stimulation of POMC neurons and inhibition of neuropeptide-Y (NPY) and Agouti-related peptide (AgRP) neurons in the arcuate nucleus [86]. Evidence supports the significant efficacy of liraglutide in reducing body mass index (BMI) in adolescents aged 12 to less than 18 years old [87]. This significant effect was pronounced after 52 weeks of treatment by achieving at least 5% reduction in BMI in 43.3% of participants in the liraglutide group in addition to 10% BMI reduction in 26.1% of liraglutide subjects compared with 18.7% and 8.1%, respectively, in control participants. However, this notable effect was not proven after 26 weeks of intervention.

In addition, it was reported that the most frequent associated adverse effects were the mild-to-moderate effects of gastrointestinal origin, mainly nausea, vomiting and diarrhea [87]. Although pancreatitis is a commonly reported adverse effect caused by liraglutide, this adverse effect was not recognized in adolescents, as studies conducted on this population did not predict the association of pancreatitis incidence and liraglutide administration except in only one participant who suffered from a single moderate episode and recovered rapidly without treatment [87]. Earlier in 2014, it was approved for adult use in obesity. In April 2020, Liraglutide 3 mg was also approved as an add-on therapy for managing obesity in adolescents aged 12–17 [88].

#### 5.1.3. Phentermine and Topiramate Combination

The combination of Phentermine and Topiramate was approved in July 2022 in the USA and is considered the newest approved add-on medication for the chronic management of childhood obesity based on the evidence from clinical trials [89]. This combination exerts a synergistic effect by acting on appetite suppression and increasing satiety, the exact anorectic effect is not fully understood. A clinical trial that examined the use of this combination in adolescents at two different dose intensities (mid- and top-doses) found that it was safe and tolerable and resulted in significant weight loss of 5% in 13.3% and 50%, respectively, of those who received the mid- and top-doses, as determined on day 56 of the treatment [90]. Table 1 shows a comparison of FDA-approved medications for pediatric obesity.

#### 5.1.4. Setmelanotide (FDA-Approved for Syndromic Obesity)

Setmelanotide is a melanocortin 4 receptor (MC4R) agonist that was approved by the FDA in November 2020 as a medication for obesity management in adults and pediatrics of 6 years and older suffering from genetic conditions proopiomelanocortin (POMC), proprotein convertase subtilisin/kexin type 1 (PCSK1), or leptin receptor (LEPR) deficiency. Later in June 2022, it received FDA approval to treat impaired hunger signaling caused by Bardet-Biedl Syndrome (BBS) [91]. Although BBS is a rare genetic disorder in which obesity is a characteristic feature, it is also characterized by retinal dystrophy, renal dysfunction, hypogonadism and learning difficulties [91]. This syndrome is caused by a genetic variation that disrupts the melanocortin pathway, which regulates body weight [92]. Setmelanotide acts through a unique mechanism by activating MC4R and overcoming genetic defects in the melanocortin pathway [92]. Several clinical trials showed that setmelanotide was associated with a statistically significant decline in body weight by 5.5% and hunger scores in individuals with BBS [93]. The most reported adverse effects are hyperpigmentation, nausea, and vomiting [94].

### 5.2. Off-Label Medications

#### 5.2.1. Metformin

Metformin is currently FDA-approved in pediatrics of 10 years of age for managing Type 2 Diabetes Mellitus (T2DM) [95]. In a systematic review conducted by Brufani et al. to investigate the impact of metformin on weight loss in obese non-diabetic adolescents, nine out of eleven included studies proved that metformin causes a slight but statistically significant reduction in BMI from 1.1 up to 2.7 compared with placebo or lifestyle intervention alone following 6 to 12 months of treatment, suggesting that metformin possesses a modest effect as an anti-obesity medication [96]. Metformin exerts this weight-reducing effect by inhibiting liver glucose production, suppressing appetite, improving insulin sensitivity and regulating fat oxidation and storage [97]. The recommended dose of metformin for weight loss is 200–500 mg, initially not to exceed 2 g/day. The most commonly reported adverse effects are gastrointestinal, such as abdominal pain, reduced appetite, diarrhea, nausea, altered taste and vomiting [94].

#### 5.2.2. Lisdexamfetamine

Lisdexamfetamine is FDA approved for the management of attention-deficit hyperactivity disorder (ADHD) in the pediatric population of age six years [98]. A retrospective review stated that lisdexamfetamine administration in adolescents between 12 and 19 years being affected by binge eating disorder (BED) resulted in a slight and insignificant reduction in the BMI percentile but prevented further weight gain [99]. Out of 25 participants, fifteen had some level of improvement of their BED symptoms described as complete remission (four cases), reduction in BED frequency (six cases), likely to binge if LDX is skipped (two cases), less frequent sneaking of food (one case), and occasional BED in response to increased stress (two cases).

#### 5.2.3. Zonisamide

Zonisamide is an antiepileptic medication approved by the FDA as an adjunct therapy in partial seizures for adolescents of 16 years and adults [100]. The addition of zonisamide to treating a 15-year-old obese male admitted to the hospital for depression and anxiety symptoms resulted in appetite suppression by acting on the hypothalamus, weight loss and normalization of his triglyceride levels [101]. In addition, results from clinical trials investigating the effect of zonisamide as add-on therapy in children suffering from partial seizures on weight reduction showed that about 35.8% of the treated patients experienced significant weight reduction (≥5% reduction of body weight) [102].

#### 5.2.4. Naltrexone S.R. with Bupropion S.R.

Naltrexone is an opioid receptor antagonist that is FDA approved for managing opioid and alcohol dependence [103]. Bupropion is a dopamine/norepinephrine reuptake inhibitor approved to be used in depression and aids in smoking cessation [104]. In a 24-week study, it was stated that the administration of naltrexone S.R. and bupropion S.R. combination has a significant weight reduction in adults with overweight and obesity by −9.4 ± 6.4 kg [20.7 ± 14.1 lb] [105]. The exact mechanism of action has not been fully described, but this combination acts central leading to appetite suppression [106]. However, its efficacy and safety have not yet been evaluated in children necessitating further research in this population.

#### 5.2.5. Fluoxetine

Fluoxetine is an approved antidepressant medication for children eight years of age and older [107]. There is good evidence that obesity is associated with depression and less consistent evidence that depression is linked to obesity [108]. Among adults with overweight or obesity suffering from depression, fluoxetine showed a modest effect on weight loss as an anti-obesity medication by achieving weight loss of 2.7 kg and a decline in body mass index by 1.1 kg/m^2^ compared with placebo, owing to the alleviation of hunger inhibition signals and thus reduced food intake [109]. However, further research is necessary to investigate the impact of this medication on weight loss in pediatrics.

#### 5.2.6. Metabolic and Bariatric Surgery

Metabolic and bariatric surgery is considered an effective and safe treatment approach in cases of extreme obesity and comorbidities when it is followed by long-term follow-up and support for the patient and family [110]. Different procedures have different body weight loss outcomes and surgery has the greatest weight loss outcome compared to pharmacotherapy through achieving a 37% change in BMI regardless of the baseline BMI value, which has a substantial effect on the metabolic complications of obesity [111]. Moreover, bariatric procedures showed significant improvement in body mass index with short-term favorable effects on mental, physical, and social outcomes among adolescents with obesity [112]. Impressively, notable remission of hypertension and improvement in typical dyslipidemia have been described following bariatric surgeries [111]. In a meta-analysis that looked at the overall impact of bariatric surgery procedures among adolescents with severe obesity, the findings showed that all procedures were associated with significant weight reductions and decreases in overall comorbidities with an acceptable complication rate [113].

## 6. Conclusions

The rise of childhood obesity is becoming an endemic health burden all around the world. An increase in the prevalence of childhood obesity, leading to a surge in complications among pediatric age groups, contributes to an escalation in the hospital admissions due to comorbidities such as CVD, asthma, and OSA. Modifiable risk factors can be a starting point for preventive measures in shared responsibility between the government (public sector), the Food Industry (private sector), community organizations, schools, health care systems, and parents. Furthermore, pharmacological management of childhood obesity is limited and only used after non-pharmacological interventions have failed or in the late stages of obesity. The American Academy of Pediatrics is currently calling for earlier intervention and a more aggressive approach for treating pediatric obesity, which includes greater use of anti-obesity pharmacotherapy and metabolic and bariatric surgery [80]. Additional FDA-approved medications, such as semaglutide and tirzepitide, are on the horizon [114], and greater acceptance of the use of metabolic and bariatric surgery for children and adolescents provides upcoming opportunities to combat this chronic, complex, multifactorial, and relapsing disease.

## Figures and Tables

**Table 1:** FDA-approved medications for pediatric obesity.

Drug Name	Orlistat	Phentermine and Topiramate Combination	Liraglutide
Pharmacological class	Lipase inhibitor	Combination of sympathomimetic and GABA_A_ receptor agonist	Glucagon-like peptide 1 (GLP-1) receptor agonists
Dose	120 mg	NA	3 mg
Frequency	Three times daily	Once daily	Once daily
Route of administration	Orally	Orally	Subcutaneous (S.C.)
Most common adverse effects	steatorrhea, flatulence, constipation, and occasionally deficit of fat-soluble vitamins.	Paresthesia, dry mouth, constipation, dysgeusia, insomnia.	Gastrointestinal (nausea, vomiting and diarrhea)
Efficacy (expressed as reduction in BMI and/or weight)	BMI reduction by 0.7 kg/m^2^	Average weight loss by 6.2 kg.	5–10% BMI reduction
